# Soil Moisture Inversion in Alfalfa via UAV with Feature Fusion and Ensemble Learning

**DOI:** 10.3390/plants15030404

**Published:** 2026-01-28

**Authors:** Jinxi Chen, Jianxin Yin, Yuanbo Jiang, Yanxia Kang, Yanlin Ma, Guangping Qi, Chungang Jin, Bojie Xie, Wenjing Yu, Yanbiao Wang, Junxian Chen, Jiapeng Zhu, Boda Li

**Affiliations:** 1 College of Water Conservancy and Hydrpower Engineering, Gansu Agricultural University, Lanzhou 730070, China; 1073324120804@st.gsau.edu.cn (J.C.); 1073323010121@st.gsau.edu.cn (Y.J.); qigp@gsau.edu.cn (G.Q.); jiebj@gsau.edu.cn (B.X.); 14760650228@163.com (W.Y.); 1073323020378@st.gsau.edu.cn (Y.W.); 1073323020360@st.gsau.edu.cn (J.C.); 1073324020383@st.gsau.edu.cn (J.Z.); 1073324020375@st.gsau.edu.cn (B.L.); 2 Zhejiang Institute of Quality Sciences, Hangzhou 310018, China; 18834171849@163.com; 3 Qingyang Hydrological and Water Resources Survey Center, Qingyang 745000, China; yanxiakang@126.com

**Keywords:** soil moisture, machine learning, ensemble learning model, multi-source feature fusion, alfalfa grassland

## Abstract

Timely access to soil moisture conditions in farmland crops is the foundation and key to achieving precise irrigation. Due to their high spatiotemporal resolution, unmanned aerial vehicle (UAV) remote sensing has become an important method for monitoring soil moisture. This study addresses soil moisture retrieval in alfalfa fields across different growth stages. Based on UAV multispectral images, a multi-source feature set was constructed by integrating spectral and texture features. The performance of three machine learning models—random forest regression (RFR), K-nearest neighbors regression (KNN), and XG-Boost—as well as two ensemble learning models, Voting and Stacking, was systematically compared. The results indicate the following: (1) The integrated learning models generally outperform individual machine learning models, with the Voting model performing best across all growth stages, achieving a maximum R^2^ of 0.874 and an RMSE of 0.005; among the machine learning models, the optimal model varies with growth stage, with XG-Boost being the best during the branching and early flowering stages (maximum R^2^ of 0.836), while RFR performs better during the budding stage (R^2^ of 0.790). (2) The fusion of multi-source features significantly improved inversion accuracy. Taking the Voting model as an example, the accuracy of the fused features (R^2^ = 0.874) increased by 0.065 compared to using single-texture features (R^2^ = 0.809), and the RMSE decreased from 0.012 to 0.005. (3) In terms of inversion depth, the optimal inversion depth for the branching stage and budding stage is 40–60 cm, while the optimal depth for the early flowering stage is 20–40 cm. In summary, the method that integrates multi-source feature fusion and ensemble learning significantly improves the accuracy and stability of alfalfa soil moisture inversion, providing an effective technical approach for precise water management of artificial grasslands in arid regions.

## 1. Introduction

Soil moisture content is a key parameter in the energy balance and hydrological cycle between the land surface and the atmosphere, and it is also an important factor limiting crop growth and development in arid and semi-arid regions [1]. Accurately estimating soil moisture can provide a reliable reference for precision irrigation [2]. Traditional methods for measuring soil moisture mainly include the neutron probe method, the drying method, and the time-domain reflectometry method. However, these methods have drawbacks such as being time-consuming, labor-intensive, and costly in sampling, making them unsuitable for regional soil moisture estimation [3]. At present, satellite remote sensing technology has partially enabled rapid large-scale monitoring of soil moisture conditions, but there are still issues such as poor timeliness, high costs, and low accuracy [4,5,6,7]. Compared with traditional surveying methods and satellite remote sensing technology, UAV remote sensing technology has advantages such as low cost, easy accessibility, high timeliness, and high spatial and temporal resolution, providing technical support for the further development of precision agriculture [8].

In recent years, with the rapid development of computer technology, machine learning algorithms [9] have become an important means of predicting soil moisture content. For example, Wang [10] and others combined machine learning algorithms to construct a UAV data-driven comprehensive moisture index model, achieving an estimation of the comprehensive moisture index for summer maize; Jin [11] and others used the Pearson correlation coefficient to screen feature variables and, combined with machine learning models, effectively improved the accuracy of soil moisture inversion. However, when the dataset is relatively small, these machine learning methods are prone to overfitting [12], whereas ensemble learning, by combining multiple machine learning models, can demonstrate stronger robustness and generalization ability when dealing with complex variables. Peppes [13] and others found that multi-model voting ensemble methods based on traditional machine learning perform well in the agricultural field; Das [14] and others used three ensemble learning methods (bagging, boosting, and stacking) to invert surface soil moisture in semi-arid areas. From the perspective of feature construction, the main method of UAV-based crop remote sensing monitoring is to obtain crop spectral information by equipping sensors, such as visible light, multispectral, and hyperspectral, and to extract information such as vegetation indices [15] for inversion. As a unique optical parameter extracted from the spectral information of vegetation canopies [16], the vegetation index has been widely used in soil moisture estimation, but its predictive efficiency and accuracy still need to be further improved [17]. This is mainly because vegetation indices primarily reflect the optical responses within crops but fail to adequately capture the external morphological information of the crops. In contrast, image data can extract morphological features such as texture—texture reflects the repeating patterns and regularity information in an image and has gradually been used for soil moisture monitoring [18]. However, relying solely on texture features to estimate soil moisture content still has limited accuracy. Therefore, integrating spectral information with image texture features is expected to compensate for the shortcomings of a single data source, thereby improving the accuracy and robustness of soil moisture retrieval [19]. At present, research on alfalfa soil moisture using multi-source feature fusion and ensemble learning models is still very limited. Most studies are based on single-feature variables and single machine learning models, making it difficult to comprehensively reflect the variation characteristics of soil moisture, and problems such as low inversion accuracy and poor generalization performance are likely to occur. Therefore, this study employs multi-source feature fusion and ensemble learning methods to invert alfalfa soil moisture.

Artificial grasslands play a key role in modern efficient livestock production systems, effectively overcoming the limitations of natural grassland productivity and reducing the pressure of traditional grazing methods on the ecological environment [20]. At the same time, through an intensive management model, it can stably provide large amounts of high-quality fodder, meet the nutritional needs of livestock, and play an important role in promoting the sustainable development of animal husbandry, maintaining ecological balance, and improving overall production efficiency. At the current stage, the use of UAV multispectral remote sensing and integrated learning inversion is mainly applied to crops such as corn and wheat, but in artificial grassland systems, research combining multi-source features (spectral, texture) with integrated learning models is still insufficient. Alfalfa has characteristics such as good adaptability, high forage yield, and rich nutrition [21,22] and is known as the ‘king of forage crops’ [23]. It also plays an important role in the development of livestock farming and ecological environmental protection both domestically and internationally [24]. Therefore, this study focuses on alfalfa, aiming to (1) clarify the response relationship between soil moisture in alfalfa fields at different growth stages and multi-source remote sensing characteristics; (2) identify the optimal feature combinations and retrieval models for each growth stage; and (3) propose a robust UAV-based remote sensing monitoring framework for soil moisture in alfalfa fields, providing a theoretical basis for precise irrigation of forage and efficient water resource management in arid and semi-arid regions.

## 2. Results

### 2.1. Characteristics of Soil Moisture Variation

The 144 samples were divided into three stages—branching (D1), budding (D2), and early flowering (D3)—with 48 sampling points in each stage. Table 1 shows that mean soil moisture was highest at 40–60 cm and lowest at 0–20 cm across all stages, indicating that soil moisture increases with depth.

**Figure 1:**
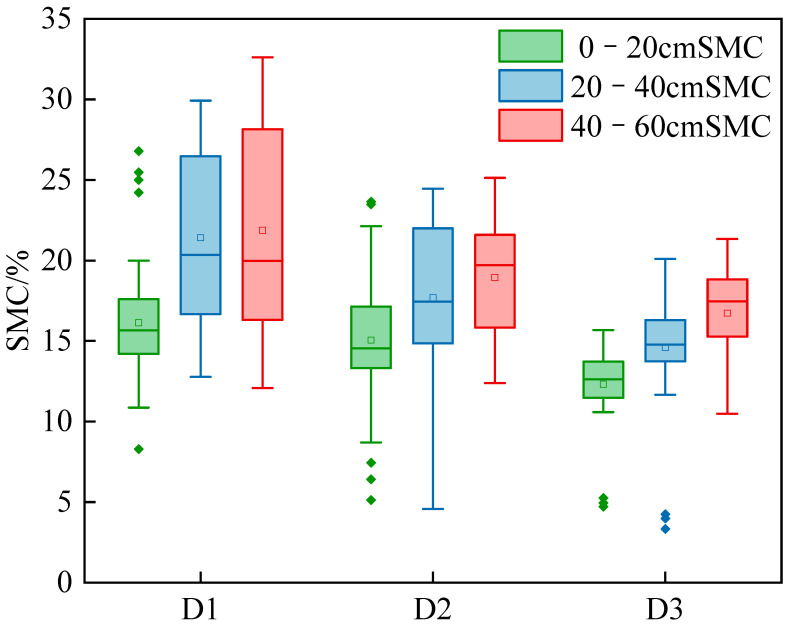
Box plot of soil moisture content changes at different growth stages of alfalfa. D1: Branching stage, D2: Budding stage, D3: Early flowering stage.

During the branching stage, the average soil moisture content was between 16.1% and 21.9%; during the budding stage, the average was between 15.1% and 18.9%; and during the early flowering stage, the average soil moisture content was between 12.3% and 16.7%. Overall, from the branching stage to the early flowering stage, the average soil moisture content showed a decreasing trend, and the degree of fluctuation gradually decreased. As shown in Figure 1, soil moisture content exhibits a gradual downward trend from the branching stage to the early flowering stage of alfalfa, and all samples strictly follow a normal distribution.

### 2.2. Study on the Correlation Between Soil Water Content and Various Variables at Different Growth Stages and Soil Depths

**Figure 2:**
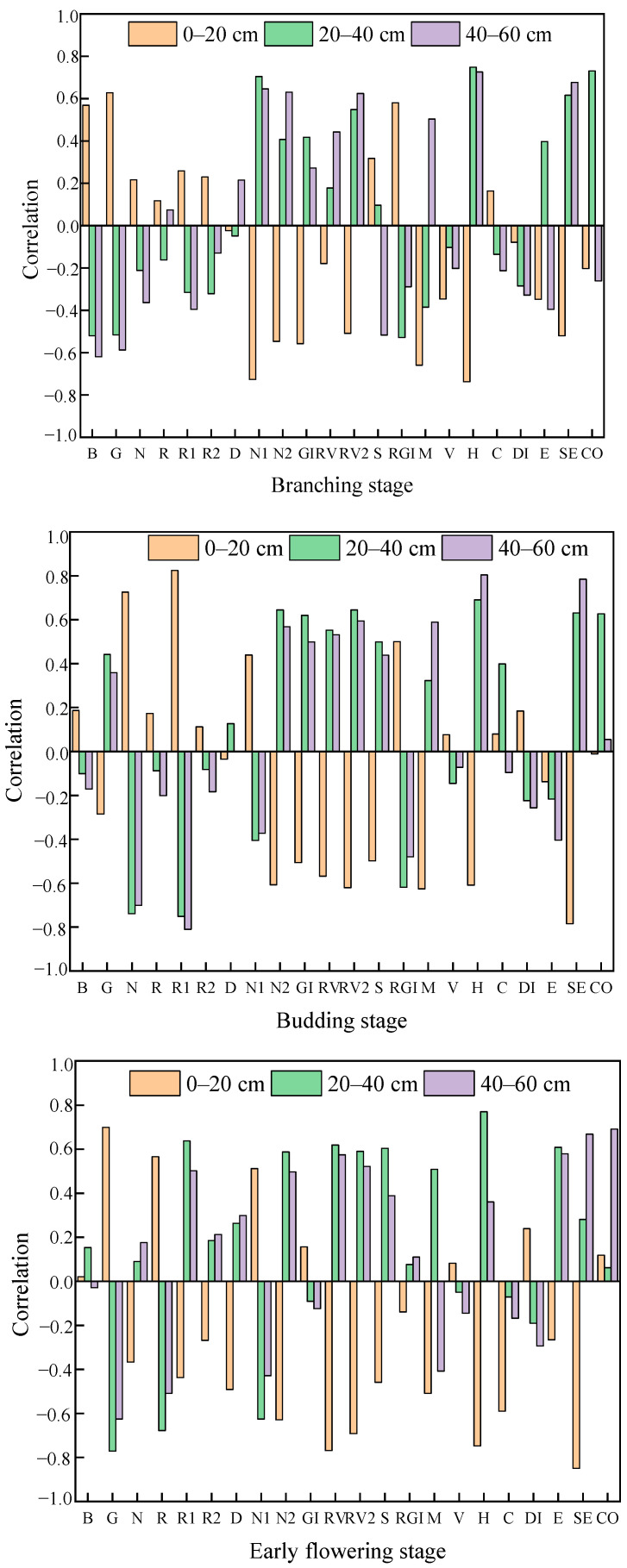
Correlation between feature variables and soil moisture. B: Blue; G: Green; R: Red; N: NIR; R1: red edge 720; R2: red edge 750; D: DVI; N1: NDVI1; N2: NDVI2; GI: GI; RV: RVI1; RV2: RVI2; S: SRPI; RGI: RGRI; M: MEA; V: VAR; H: HOM; C: CON; DI: DIS; E: ENT; SE: SEC; CO: COR.

A Pearson correlation analysis was conducted on the soil moisture content at different soil depths and the spectral reflectance, spectral indices, and texture features after removing the soil background for each growth stage (branching stage, bud stage, and early flowering stage). It was found that the correlation at the 0–20 cm depth was relatively weak, possibly because this soil layer is more sensitive to recent irrigation or evaporation, causing rapid changes in surface moisture and thus weaker correlations. Based on this, a systematic analysis was carried out to select the optimal feature variable combinations for each growth stage and soil depth (Figure 2), providing key inputs for subsequent modeling. During the branching stage of alfalfa, at the 0–20 cm depth, the variables most strongly correlated with soil moisture content were Blue, Red, NDVI1, GI, RGRI, MEA, HOM, and SEC, with correlation coefficients all greater than 0.55; at the 20–40 cm depth, the variables with strong correlations included Blue, Red, NDVI1, RVI2, RGRI, HOM, SEC, and COR. At the 40–60 cm depth, Blue, Red, NDVI1, NDVI2, RVI2, MEA, HOM, and SEC all exhibited strong correlations. At the current budding stage, at a depth of 0–20 cm, Red, Green, NDVI2, RVI1, RVI2, MEA, HOM, and SEC are most closely related to soil moisture, with most correlation coefficients exceeding 0.6; at a depth of 20–40 cm, the main variables are Red, Green, NDVI1, GI, RVI2, HOM, SEC, and COR, with all correlation coefficients above 0.6. At a depth of 40–60 cm, the primary related variables are Red, Green, NDVI2, RVI1, RVI2, MEA, HOM, and SEC. By the early flowering stage, at a depth of 0–20 cm, the correlation coefficients of Red, Green, NDVI2, RVI1, RVI2, HOM, CON, and SEC were all higher than 0.55. At a depth of 20–40 cm, the variables with significant correlations were Red, Green, NDVI1, RVI1, SRPI, MEA, HOM, and ENT. At a depth of 40–60 cm, Green, Red, NDVI2, RVI1, RVI2, ENT, SEC, and COR were predominant.

### 2.3. The Advantages of Ensemble Learning and Multi-Source Data Fusion

#### 2.3.1. The Advantages of Ensemble Learning

**Figure 3:**
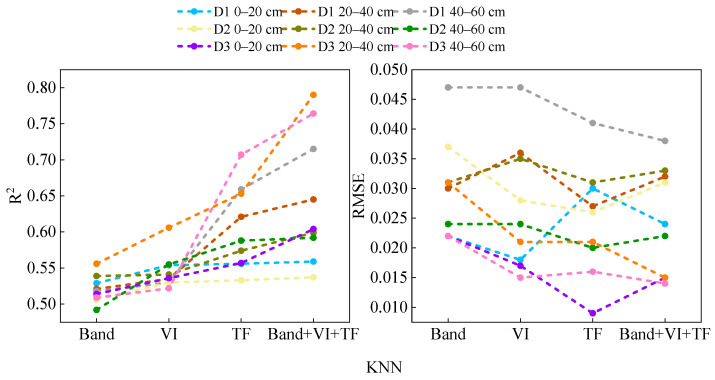

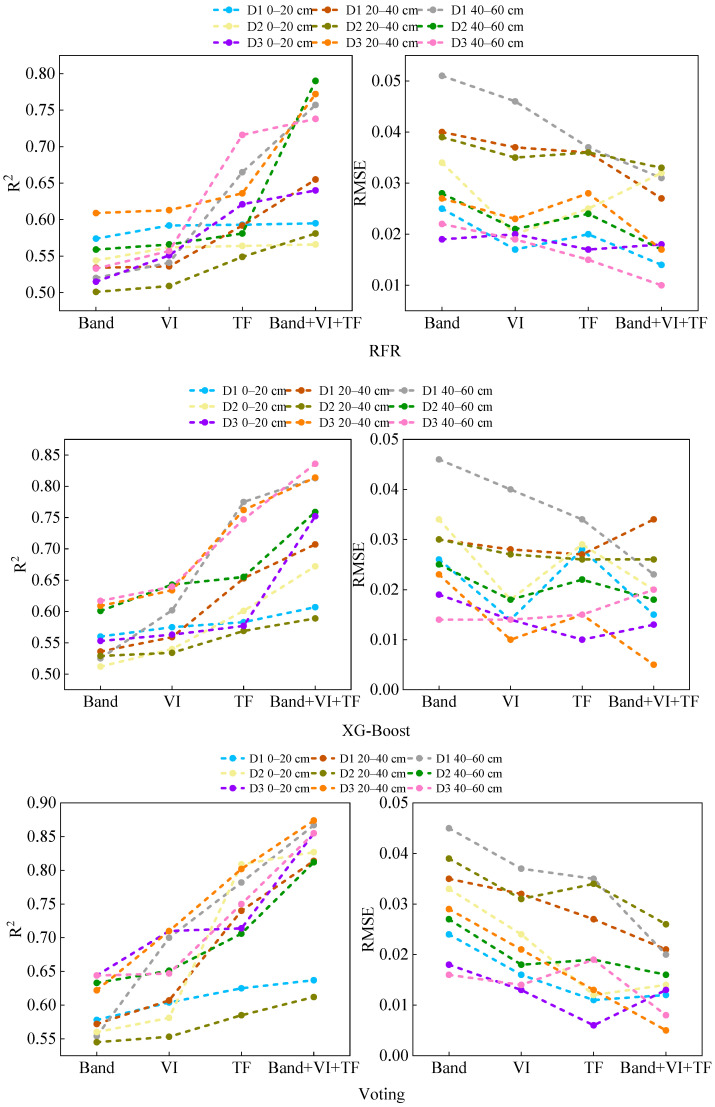

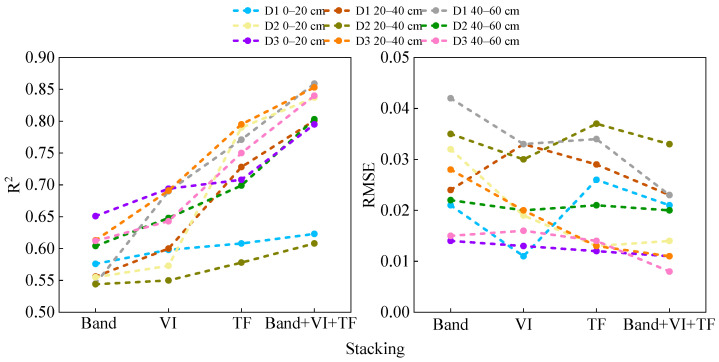
Soil moisture estimation performance of various models and different types of input features.

To assess the potential of ensemble learning methods in improving the accuracy of soil moisture inversion, this study input different sets of feature variables into three individual machine learning models and two ensemble learning models for comparative analysis. As shown in Figure 3, throughout the entire growth period of alfalfa, under different soil depth conditions, when individual feature variables (spectral reflectance, spectral indices, and texture features) were used as inputs, the models corresponding to texture features generally achieved the highest accuracy. Specifically, during the branching stage, the XG-Boost model achieved a maximum *R*^2^ of 0.775 with a root mean square error (RMSE) of 0.034; the RFR model had a maximum *R*^2^ of 0.665 with an RMSE of 0.037; and the KNN model reached a maximum *R*^2^ of 0.659 with an RMSE of 0.041. The Voting and Stacking ensemble models achieved maximum *R*^2^ values of 0.782 and 0.771 during this stage, with corresponding RMSEs of 0.035 and 0.034, respectively. At the current budding stage, the XG-Boost model had an *R*^2^ of up to 0.655 and an RMSE of 0.022; the RFR model had an *R*^2^ of 0.581 and an RMSE of 0.024; and the KNN model had an *R*^2^ of 0.588 and an RMSE of 0.020. In comparison, the Voting and Stacking models performed better, with *R*^2^ values of 0.809 and 0.790, and RMSE values of 0.012 and 0.013, respectively. By early flowering stages, the *R*^2^ of the XG-Boost model reached a maximum of 0.762, with an RMSE of 0.015. The *R*^2^ of the RFR model was 0.636, with an RMSE of 0.028; the *R*^2^ of the KNN model was 0.653, with an RMSE of 0.021. The Voting and Stacking models still maintained high accuracy, with *R*^2^ values of 0.802 and 0.795, respectively, and RMSEs both at 0.013. These results indicate that ensemble learning methods can effectively improve the inversion accuracy of soil moisture across different growth stages.

#### 2.3.2. Advantages of Multi-Source Feature Integration

To improve the accuracy and robustness of soil moisture estimation, this study breaks through the traditional single-feature inversion approach and proposes a modeling method based on the fusion of multi-source remote sensing features. The soil moisture estimation results under different feature combinations are shown in Figure 3. When using a single feature as input, there are significant differences in the performance of the models: with texture features as input, *R*^2^ ranges from 0.533 to 0.802, whereas for models based on spectral indices and spectral reflectance, *R*^2^ values are in a lower range of 0.492 to 0.643, with RMSE all below 0.05. The results indicate that texture features demonstrate more stable and superior estimation capabilities among the various types of input.

**Figure 4:**
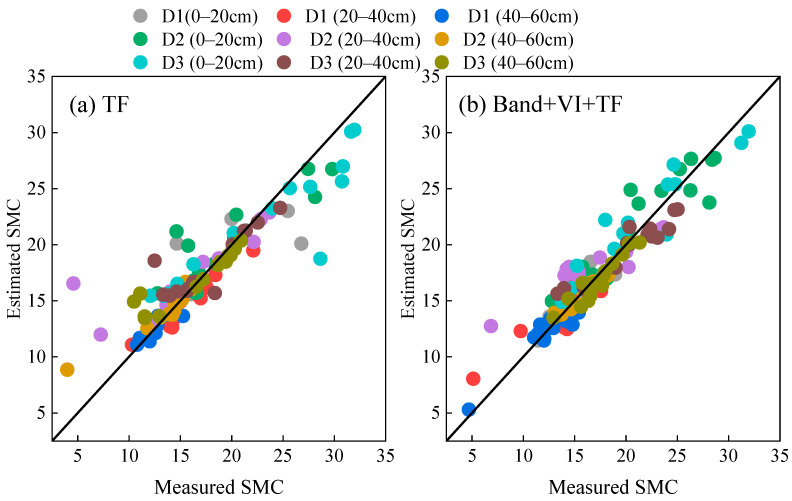
Prediction performance of Voting on soil moisture content of different feature types.

A comparison of the results of inverting alfalfa soil moisture using texture features (TF) and multi-source feature fusion (Band + VI + TF) as input variables with the Voting model is shown in Figure 4. When using only texture features, the scatter points show significant dispersion around the 1:1 line, reflecting considerable fluctuations in the estimated results. In contrast, the scatter points for multi-source feature fusion (Band + VI + TF) are more closely distributed near the 1:1 line, indicating higher consistency and fitting accuracy. The fusion of multi-source features combines band reflectance, spectral indices, and texture information, statistically demonstrating better and more robust inversion performance.

#### 2.3.3. Optimal Soil Depth and Estimation Model

**Figure 5:**
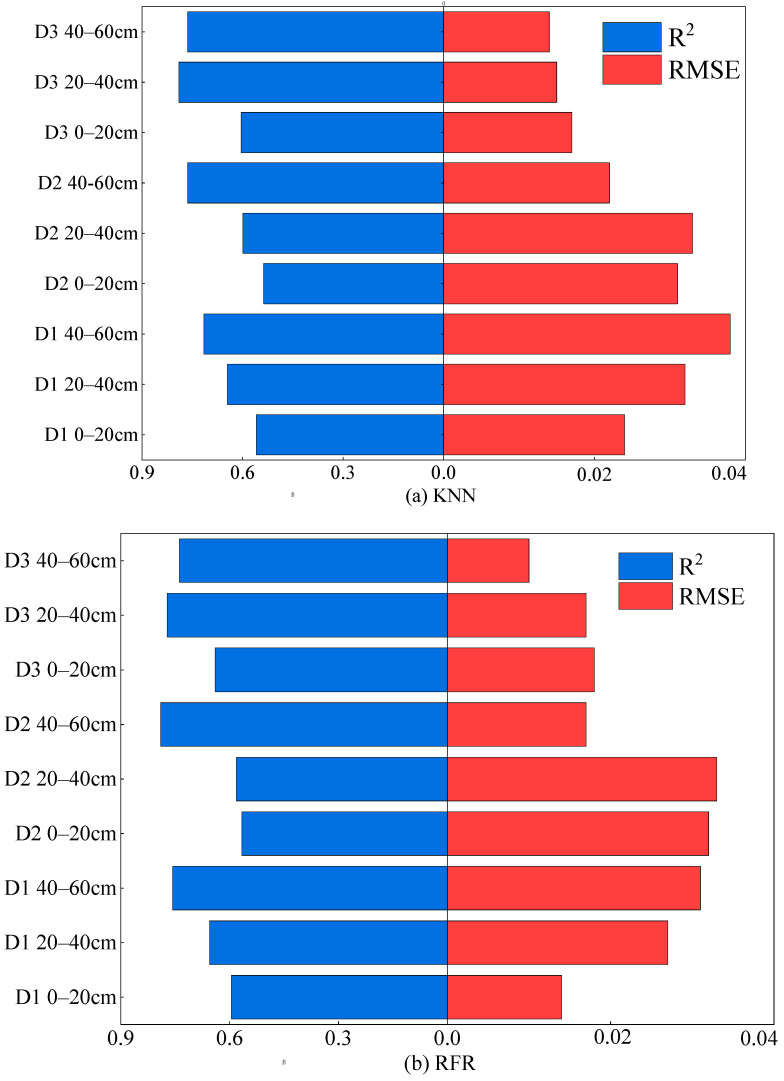

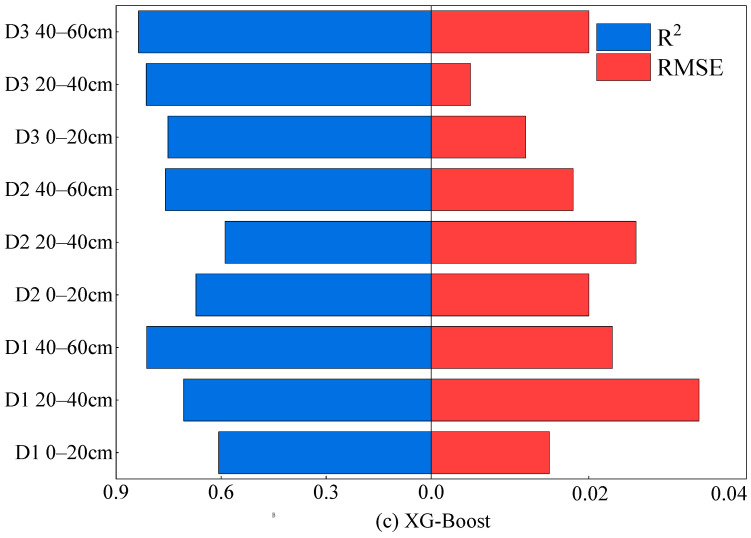
Comparison of Machine Learning Model Accuracy. In (**a**) KNN model; (**b**) RFR model; (**c**) XG-Boost model.

Based on the optimal feature variable obtained in Section 2.3.2 (Band + VI + TF), this study therefore uses Band + VI + TF as the independent variable in the machine learning models, with SMC as the dependent variable. KNN, RFR, and XG-Boost are applied to different depths at three different time periods (Figure 5).

During the branching period, the accuracy of the RFR, KNN, and XG-Boost models all increased with soil depth, and the accuracy of XG-Boost was generally higher than that of the KNN and RFR models. The highest *R*^2^ of XG-Boost was 0.813, with an RMSE of 0.023. The *R*^2^ value at 40–60 cm is closer to 1, indicating that the model is more stable at this depth, and all three models reach their maximum at 40–60 cm. Therefore, during the branching stage, 40–60 cm is the optimal inversion depth. During the budding stage, the prediction accuracy of both the KNN and RFR models increases with the depth of the soil layer, while the RMSE values show the opposite trend. Therefore, the optimal inversion depth during the bud stage is also 40–60 cm. During the early flowering stage, both the KNN and RFR models reached their maximum values at 20–40 cm, with *R*^2^ values of 0.790 and 0.772 and RMSE values of 0.015 and 0.017, respectively. Therefore, 20–40 cm was determined to be the optimal inversion depth for the early flowering stage.

By comparing the accuracy of each model on the validation set, during the branching stage and the early flowering stage, the XG-Boost model outperformed KNN and RFR models at all depths, with a maximum *R*^2^ of 0.813 and an RMSE of 0.023. During the bud stage, the RFR model reached the highest performance, with an R^2^ of 0.790 and an RMSE of 0.017. Therefore, XG-Boost was determined to be the best inversion model.

### 2.4. Soil Moisture Distribution by Depth Across Growth Stages

**Figure 6:**
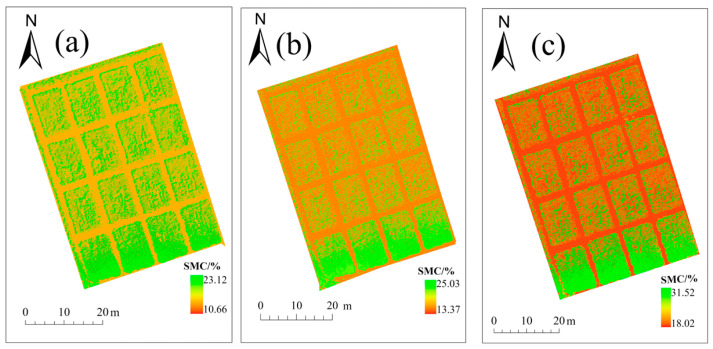

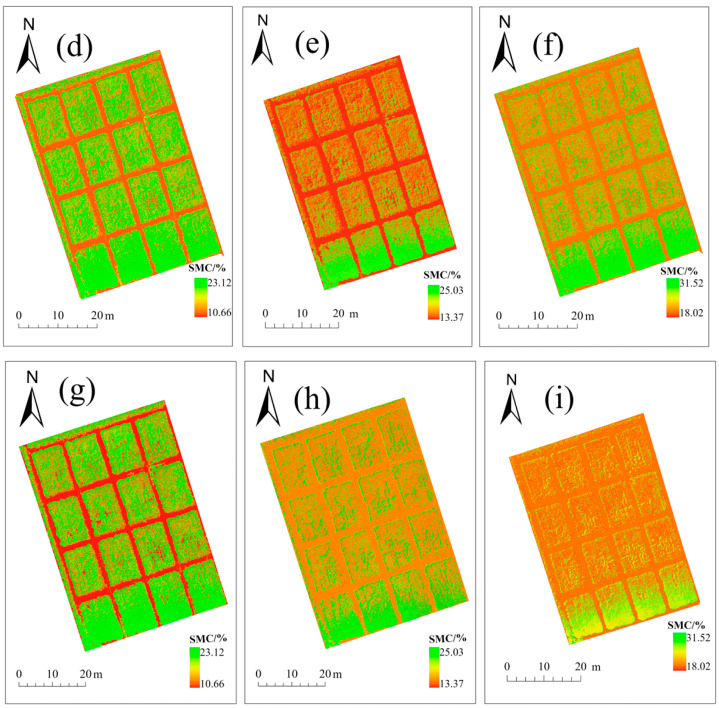
Spatial maps of soil moisture at various depths across the study’s growth stages. (**a**–**c**) show the spatial distribution of soil moisture at 0–20 cm, 20–40 cm, and 40–60 cm depths during the branching stage of alfalfa; (**d**–**f**) show the spatial distribution of soil moisture at the same depths during the bud stage of alfalfa; (**g**–**i**) show the soil moisture distribution at the same depths during the early flowering stage of alfalfa.

Based on the optimal soil moisture inversion model obtained in Section 2.3, the soil moisture at three different depths was inverted using these models, resulting in soil moisture distribution maps for each depth throughout the entire process (Figure 6). As shown, the inverted soil moisture distribution maps indicate that, across the three growth stages, moisture gradually increases from the surface to deeper layers; the maximum value reached 31.52%. Soil moisture during the branching and budding stages is generally higher than during the early flowering stage. This finding is consistent with measured soil moisture data, confirming the reliability of the results. The inversion maps of different growth periods and soil layers were all completed in ArcGIS Pro.

## 3. Discussion

### 3.1. Effect of Phenological Stages on Soil Moisture Retrieval Accuracy

This study found that the spectral features sensitive to soil moisture vary at different growth stages. This may be because the factors affecting the reflectance changes in green plant leaves differ significantly in the visible and near-infrared spectral regions. In the visible spectral region, pigments such as chlorophyll and carotenoids play a dominant role in vegetation spectra, with chlorophyll being particularly crucial. In the near-infrared spectral region, the spectral characteristics of plants are mainly influenced by the internal cellular structure of the leaves, with multiple reflections between the cell walls and cell spaces of the leaves leading to a high reflectance in the near-infrared spectrum [25,26,27,28,29]. Therefore, the vegetation indices constructed based on different spectral bands show significant differences in response to water stress throughout the growing season. Thus, the vegetation indices selected at different growth stages exhibit considerable variation in their sensitivity to water stress. When using vegetation indices alone, spectral reflectance alone, or vegetation indices combined with spectral bands as feature variables, most models perform better in the branching and early flowering stages but relatively poorly in the budding stage. The reason may be that during the branching stage, alfalfa is in a rapid growth phase, requiring a large amount of nutrients and water. Therefore, soil moisture during this growth stage has a significant impact on vegetation indices and spectral reflectance, resulting in better model inversion performance. During the budding stage, however, the relatively higher rainfall may lead to little difference in soil moisture among plots, which reduces inversion accuracy. In the early flowering stage, the vegetation has already reached full coverage, minimizing the influence of soil pixels. Soil moisture directly affects vegetation indices, resulting in higher inversion accuracy at this stage.

### 3.2. Analysis of Soil Moisture Estimation Based on Multi-Source Feature Fusion

Combining texture features with spectral features can effectively improve the accuracy of soil moisture estimation by the model. This may be because when using vegetation indices derived from spectral information to estimate crop traits, sensors are often affected by the problem of spectral saturation [30], making it difficult to accurately measure spectral information, which is more common in crops with high canopy density [31]. Texture features can effectively characterize the spatial structure and geometric morphology of crop canopies [32], as they are analyzed based on the spatial variation in pixel grayscale, making them relatively insensitive to spectral saturation [33]. However, these features are significantly influenced by vegetation growth status and environmental conditions [34]. Under vegetative cover, texture information mainly comes from canopy structure rather than the soil surface. Due to the lack of leaf area index (LAI) and plant height data, it is difficult to quantify the contribution of vegetation growth and canopy structure changes to texture features, which may result in inversion models being unable to effectively distinguish between vegetation structure effects and soil moisture signals, thereby affecting inversion accuracy. By combining spectral features with texture features, a multidimensional dataset can be formed, reducing the limitations, errors, and uncertainties that may arise from a single data source [35,36,37]. Different spectral indices and texture indices have varying adaptability to environmental conditions. Through multi-source feature fusion, the model’s adaptability and accuracy under different environmental conditions can be further improved. Spectral indices are mainly based on the reflective characteristics of vegetation in the infrared and visible light bands, reflecting the real-time physiological growth status of vegetation and indicating the level of photosynthetic activity. In contrast, canopy texture features involve the structural information of crop leaves. By combining these two types of features, the model can more accurately capture the complex relationships between soil moisture, vegetation growth status, and soil structure. Therefore, combining these two features can complement each other’s shortcomings and improve the model’s accuracy in estimating soil moisture content. The results of this study are consistent with the conclusions reported by Li [38], indicating that multi-source feature fusion can significantly improve inversion accuracy, outperforming single feature variables; Li [39] and others also confirmed that multi-source feature fusion can effectively enhance model prediction performance. In addition, Sun [40] and colleagues further verified the significant promoting effect of multi-source feature fusion on estimation model accuracy by using texture features of band images obtained through the combination of vegetation indices and principal component analysis as input variables.

### 3.3. The Importance of Machine Learning and Ensemble Learning in Soil Moisture Prediction

Different modeling methods significantly affect the predictive accuracy of models. This study used three machine learning algorithms to construct soil moisture prediction models. The research found that when vegetation indices were included in spectral reflectance, the XG-Boost model showed higher accuracy compared to the KNN and RFR models. This may be because XG-Boost excels at handling nonlinear relationships and complex data patterns, allowing it to better fit the complex relationship between soil moisture and input features [41]. In comparison, KNN and RFR models may be less flexible than XG-Boost when dealing with high-dimensional, nonlinear data, so their prediction accuracy may be relatively lower. Moreover, XG-Boost improves the model’s generalization ability by optimizing the loss function, allowing it to perform well outside the training set. This indicates that the XG-Boost model can better adapt to new datasets and perform more consistently on the test set, thereby improving model accuracy [42]. XG-Boost can also automatically select the most important features and has good handling capabilities for missing and outlier values, which helps reduce the model’s sensitivity to noise and unnecessary features, thereby improving model accuracy [43].

Although individual models like RFR and XG-Boost perform well at specific growth stages, their predictive ability varies depending on the dataset used. Ensemble methods can enhance the stability of model predictions and the accuracy of the results by leveraging multiple models [44]. This study used two ensemble learning models, and the results show that the ensemble learning models significantly improved the prediction accuracy of soil moisture content. The Voting model achieved a maximum R^2^ of 0.874, outperforming individual machine learning models. This finding is consistent with previous studies highlighting the effectiveness of ensemble models in agricultural applications. Feng et al. [45] found that when predicting alfalfa yield based on UAV hyperspectral images, ensemble learning models significantly improved prediction accuracy, with an R^2^ of 0.874. Ge et al. [46] also found that when comparing the performance of machine learning models and ensemble models in predicting rice at different growth stages, the ensemble models improved overall accuracy by 1–5%, with the Voting model performing the best in predictions. The above research indicates that the Voting algorithm not only has strong resistance to interference and overfitting but also exhibits high tolerance to soil background noise and outliers. The inversion accuracy of the Stacking model is second only to Voting, with a maximum R^2^ of up to 0.859, which is also superior to basic machine learning models. Compared to machine learning models, stacking models have lower computational complexity. Each base learner can be trained in parallel and then combined for ensemble prediction, resulting in higher computational efficiency. When resources are limited or fast model iteration is required, stacking models have a clear advantage, thus supporting applications in real-time monitoring or decision support systems in the agricultural field.

The above research indicates that ensemble learning generally has good generalization capability. Since ensemble learning models combine the strengths of multiple machine learning models, they can maintain high performance across different datasets and application scenarios, largely addressing the problem of overfitting [47]. Ensemble learning can also flexibly integrate different types of models, both linear and nonlinear, making it more adaptable and flexible when facing complex problems. With its advantages of improving accuracy, enhancing robustness, boosting generalization ability, and flexible model integration, ensemble learning has become an indispensable and important method in modern machine learning.

### 3.4. Limitations and Prospects

Soil moisture strongly influences crop development and yield. This study assesses how different crop growth stages affect soil moisture monitoring, using multi-source data fusion and ensemble learning methods. However, it has limitations: the analysis is restricted to parts of Jingtai, Baiyin (Gansu), and concentrates on alfalfa growth from May to September 2025. The model still needs validation across other years, regions, and climatic conditions. Future work should extend to additional vegetation types, climate zones, and time periods to enhance the model’s generalizability. Other environmental factors can also influence soil moisture prediction. For example, in a strong wind environment, the image collection task was completed on the sampling day. Strong winds can cause drones to shake, affecting the clarity of images captured by sensors and resulting in blurriness or out-of-focus shots. Blurry images cannot provide accurate spectral information, thereby affecting the accuracy of soil or vegetation inversion. Likewise, UAV remote sensing is vulnerable to weather: cloud cover can destabilize spectral signals, change band reflectance, and shift image tones. Thick cloud cover can reduce light intensity, creating shadowed areas. This makes it difficult to accurately monitor surface features that are shaded (such as vegetation, soil, etc.), thereby affecting the quality of the retrieved data. The presence of clouds may weaken the received spectral signal, leading to inaccurate spectral reflectance during retrieval. Variations in lighting conditions modify reflected intensities across bands and can thus degrade model prediction accuracy. In future research, strategies should be developed to determine the optimal times for shooting, selecting suitable times for observation based on weather forecasts and real-time data (such as wind speed and cloud thickness), while minimizing the impact of environmental factors. Third, the sample size of the model may affect the fitting performance, and a limited sample size can easily lead to overfitting. Overfitting is a common problem in machine learning models and ensemble learning during inversion tasks. When a model overfits the sample features in the training data, its generalization ability significantly decreases, leading to reduced inversion accuracy. To improve the robustness and generalization performance of the model, one can increase the number of high-quality samples to more comprehensively cover the data distribution or introduce methods such as cross-validation and ensemble strategies to effectively constrain model complexity, thereby enhancing its stability and predictive accuracy in complex inversion scenarios. Fourth, monitoring soil moisture content through multispectral remote sensing can be influenced by changes in crop chlorophyll and leaf area. Changes in crop leaf chlorophyll can be reflected through variations in canopy reflectance, while texture features highlight the shapes and sizes of various objects in the image. Due to the angle of illumination, many leaf shadows remain on the ground, causing texture features to have errors in reflecting crop leaf area index. At this time, combining spectral features with texture features may introduce some errors in the model, affecting the accuracy of soil moisture retrieval. Future research could integrate thermal infrared data, phenological characteristics, canopy structure, and other features to improve the accuracy of soil moisture retrieval.

## 4. Materials and Methods

### 4.1. Study Area and Experimental Design

**Figure 7:**
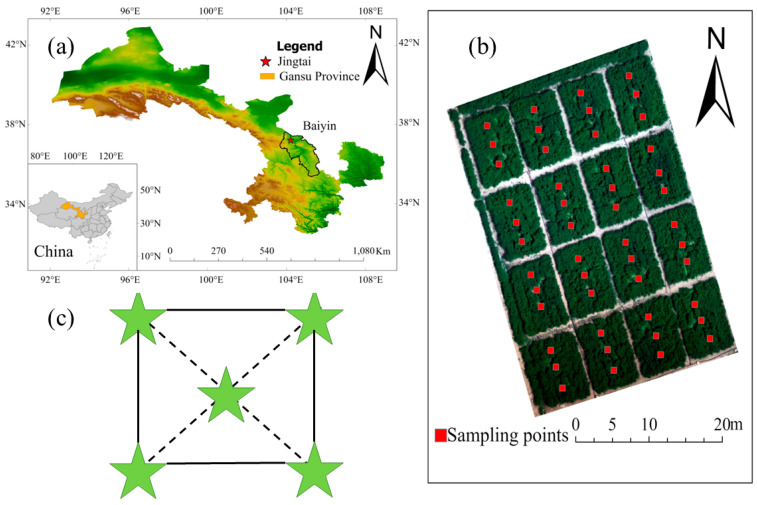
Study site location and spatial arrangement of sampling points. In (**a**) Geographical Location of the Study Area; (**b**) Sampling Point Distribution; (**c**) Five-point method. In (**c**), the pentagons represent soil sampling points; the lines indicate the outline of the sampling area.

The experiment was carried out from April to October 2025 at the irrigation experimental station of the Chuan Electric Pumping Water Resource Utilization Center in Jingtai, Gansu (37°12′59″ N, 104°05′10″ E; mean elevation 1572 m; see Figure 7). The site has a temperate continental arid climate with abundant sunshine and limited rainfall. Long-term climate parameters are as follows: annual sunshine 2652 h, frost-free period 191 d, cumulative radiation 6.18 × 10^5^ J·cm^−2^, and annual evaporation 2761 mm. Soil is loam, with mean dry bulk density 1.35 g·cm^−3^, field water-holding capacity 24.1% (mass), and pH 8.11. Topsoil nutrient contents were the following: organic matter 6.15 g·kg^−1^; total N 1.58 g·kg^−1^; total P 1.36 g·kg^−1^; total K 34.16 g·kg^−1^; available N 74.22 mg·kg^−1^; available P 32.99 mg·kg^−1^; and available K 147.80 mg·kg^−1^. A compact Davis agricultural weather station at the site recorded meteorological data. During the trial, total precipitation was 276 mm, and the mean daily temperature was 18.8 °C.

Alfalfa (Gan Nong No. 3) was used in the study. The experimental area measured 27 m (east–west) by 40 m (north–south) and was divided into 16 plots of 54 m^2^ each (6 × 9 m) to improve the inversion model’s applicability in heterogeneous sown grasslands. Plots were arranged in a completely randomized design with four irrigation levels targeting 45–55%, 55–65%, 65–75%, and 75–85% of field capacity (θf, soil water content relative to field capacity). Irrigation begins when the soil water content drops to the lower limit of the field capacity percentage, corresponding to each treatment, and continues until it reaches the upper limit of the field capacity percentage for each treatment.

### 4.2. Data Collection and Processing

#### 4.2.1. Drone Data

**Figure 8:**
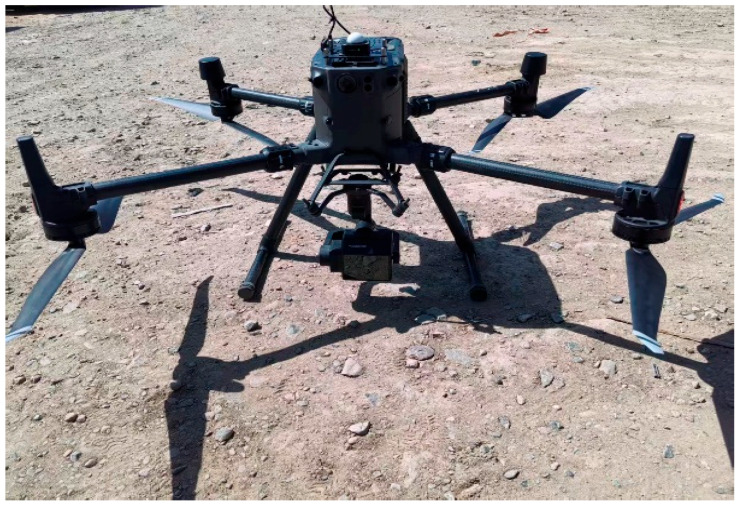
DJI Matrice 300 RTK quadcopter drone.

**Figure 9:**
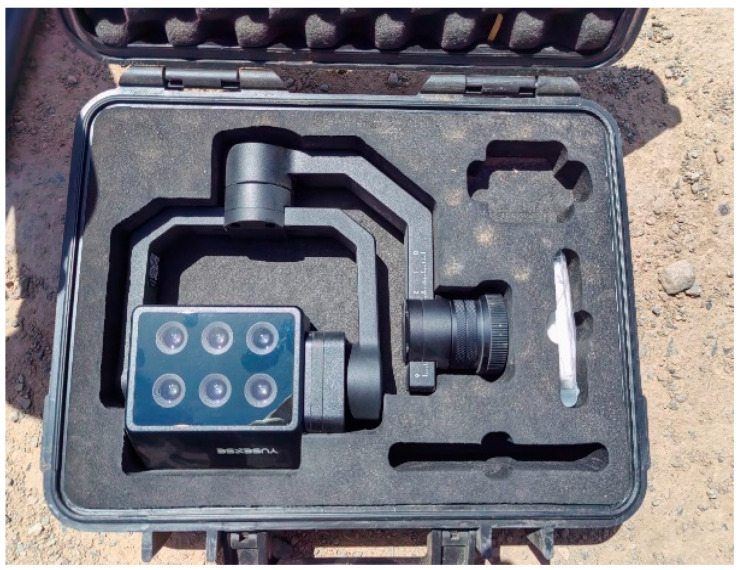
The MS 600 Pro multispectral camera.

In clear, windless, and well-lit weather, a DJI Matrice 300 RTK quadcopter drone (Figure 8) was used to collect multispectral remote sensing images (DJI Innovations, Shenzhen, China). Data were acquired on 20 May, 6 June, and 11 July 2025, corresponding to the branching, budding, and early flowering stages. The multispectral system comprises six independent 1.2-MP CMOS sensors, configured to record six bands: blue (450 nm), green (555 nm), red (660 nm), red edge 1 (720 nm), red edge 2 (750 nm), and near-infrared (840 nm) (Figure 9). The multispectral images of six bands were processed using Pix4D Mapper software, and radiometric calibration was carried out using the diffuse reflectance panels and reflection coefficients corresponding to each band, thereby generating accurate crop reflectance information. Flights were flown at 30 m altitude with 80% forward and 70% side overlap, at 2.7 m/s, between 12:00 and 13:00, with the camera oriented nadir and following preplanned routes. Table 2 lists each band’s central wavelength, bandwidth, and the reflectance of the diffuse panels. Images of ground calibration panels (ground sampling distance 2.16 cm) were taken before and after every flight to compensate for illumination changes and ensure consistency.

#### 4.2.2. Soil Moisture Content

Multispectral remote sensing images of the study area were obtained using drones, and 48 soil sampling points were selected simultaneously within 16 ground sample areas for stratified collection. The sampling points were evenly distributed across the study area (Figure 7b), and the sampling points remained fixed across the three growth stages, with soil collected at depths of 0–20 cm, 20–40 cm, and 40–60 cm. The five-point sampling method was used at each sampling point (Figure 7c).

**Figure 10:**
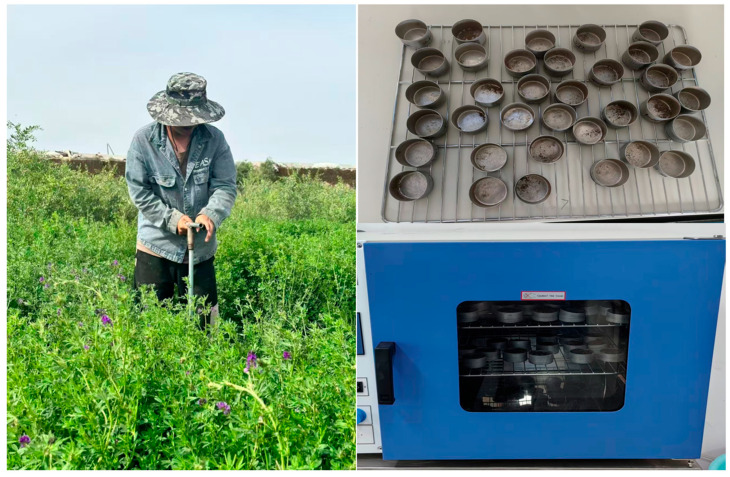
Field experiment.

When sampling, delineate a representative square area around the sampling point, and collect about 100 g of soil from the center of the area, placing it into a pre-labeled aluminum box. After sampling, determine the soil moisture content using the drying method, and take the average of five measurements as the actual soil moisture content of the sampling point (Figure 10).

#### 4.2.3. Drone Image Preprocessing

Four ground control points (GCPs) were located within the study area, and precise coordinates were determined using Real-Time Kinematic (RTK) positioning technology. Pix4D Mapper 4.8.0 was used for image alignment and georeferencing, combined with manually marked control points to improve positional accuracy. The final orthophoto map achieves a root mean square error (RMSE) of 0.23 m on the x-axis, 0.31 m on the y-axis, and 0.27 m on the z-axis. To eliminate soil background interference, the soil background was removed in ArcGIS Pro 3.4 using the vegetation index threshold method, followed by radiometric correction. We used preprocessing to convert digital numbers (DN) to reflectance. We used established reflectance calibration images to ensure spectral consistency across all bands. In addition, the MS 600 Pro multispectral sensor (Long Guang Yuchen Information Technology Equipment Co., Ltd., Qingdao, China) provides calibration coefficients to compensate for spectral response variations across six bands (blue, green, red, red edge 1, red edge 2, and near-infrared), using standard reflectance panels for field radiometric calibration to account for illumination fluctuations during UAV flight missions.

### 4.3. Calculation of Spectral Indices and Texture Features

#### 4.3.1. Spectral Index

The spectral index is a method of extracting land cover information by using the ratio or difference between certain bands in remote sensing data. In remote sensing data processing, spectral indices are widely used to extract information about vegetation, soil, water bodies, and other land cover types. This paper extracts the spectral reflectance information of sampling points based on ENVI Classic 5.3 software and calculates spectral indices using the spectral reflectance. Based on existing research results, six classic spectral indices are selected (Table 3).

#### 4.3.2. Texture Features

Texture features are a type of visual characteristic that reflect homogeneous phenomena in an image, representing the arrangement attributes of an object’s surface structure with slow or periodic changes. This study uses the gray-level co-occurrence matrix (GLCM) to statistically analyze image texture features [56]. The red light band in the vegetation canopy spectrum has a high correlation with soil moisture content and responds significantly to changes in soil moisture. Therefore, the red light band is selected to calculate the texture of multispectral images. The multispectral images of the sampling points are input into ENVI 5.3 software, and the second-order statistical filter tool is used to calculate eight feature parameters of the gray-level co-occurrence matrix for the red light band: Mean (MEA), Variance (VAR), Homogeneity (HOM), Contrast (CON), Dissimilarity (DIS), Entropy (ENT), Second Moment (SEC), and Correlation (COR).

### 4.4. Pearson Correlation Analysis

Remote sensing images usually contain multiple bands (such as visible light, near-infrared, shortwave infrared, etc.). Using all bands directly can lead to data redundancy and increased computational complexity, so Pearson correlation analysis is used to select sensitive spectral indices and reflectance combinations [57]. Pearson correlation analysis quantifies the linear relationship between two variables, with the core metric being the Pearson correlation coefficient, which ranges from −1 to 1. A coefficient of 1 indicates a perfect positive correlation, −1 a perfect negative correlation, and 0 no linear relationship; the larger the absolute value of the coefficient, the stronger the linear association between the predictor and the target variable. Common interpretation criteria are as follows: |r| ≥ 0.8 (high correlation); 0.5 ≤ |r| < 0.8 (moderate correlation); 0.2 ≤ |r| < 0.5 (low correlation); |r| < 0.2 (basically uncorrelated) [58].

### 4.5. Modeling Methods and Evaluation

#### 4.5.1. Machine Learning

This study used five machine learning algorithms, divided into basic models (K-nearest neighbors regression, random forest regression, and XG-Boost), and ensemble methods (Voting and Stacking). These five machine learning algorithms are implemented using Python 3.9’s sklearn library, with specific steps including data loading and preprocessing, data splitting, and model evaluation. Hyperparameter optimization for KNN, RFR, XG-Boost, and RR was performed using grid search combined with 4-fold cross-validation, selecting the optimal parameters based on the maximum mean R^2^. The 4-fold cross-validation allowed for a more thorough use of the data and provided a more reliable assessment of the model’s generalization ability, helping to prevent overfitting. Performance differs across models because of their algorithmic characteristics. Random forest regression constructs and aggregates many decision trees to handle complex, nonlinear relationships. KNN is an instance-based, nonparametric method that predicts outcomes from the nearest neighbors and works well with irregular data distributions. XG-Boost (Extreme Gradient Boosting) is an efficient, regularized gradient-boosting implementation designed for high speed and strong performance on large, complex datasets. Voting aggregates base model outputs (by majority or averaging), whereas Stacking trains a meta-learner on base models’ predictions to improve overall generalization (KNN’s core hyperparameter n_neighbors is 3; RFR is configured with 100 decision trees, a maximum depth of 10; random seed is 2; XG-Boost is configured with 100 decision trees, a maximum depth of 6, learning rate of 0.1).

#### 4.5.2. Ensemble Learning

Stacking is an ensemble learning method aimed at improving overall performance by combining the prediction results of multiple different models. This approach first trains several base learners on the same dataset and uses their output results as features for a subsequent meta-learner. The meta-learner is then responsible for integrating the predictions of these base models to generate the final prediction.

**Figure 11:**
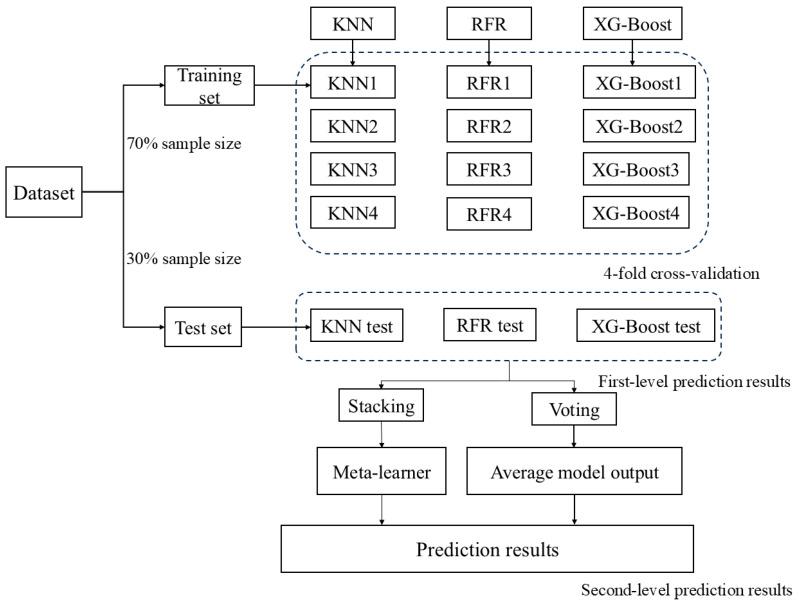
Soil moisture retrieval ensemble learning process.

The core of Voting is that different base machine learning models may have strengths in certain specific aspects, and Voting improves overall performance by combining the predictions of these models. In a Voting ensemble, all base machine learning models are independently trained and make predictions on the full training set, and the final prediction is determined by averaging the outputs of all models. Its advantages lie in its simplicity and comprehensiveness, making the final predictions more stable. To construct a Voting ensemble learning model, KNN, RFR, and XG-Boost algorithms are first used to build the first-level prediction models; then, the prediction outputs of these models are used as new features, and in the second level, the Voting strategy is applied for integration. Fiinally, by averaging the results of each model, a stable inverted soil moisture value is obtained (Figure 11).

#### 4.5.3. Evaluation Metrics

A total of 144 samples were collected over three reproductive stages. For each stage, samples were randomly split into 70% for training and 30% for testing. Model performance was assessed by R^2^ (coefficient of determination; values closer to 1 indicate better agreement between predictions and observations) and RMSE (root mean square error; lower values indicate smaller average prediction errors).

### 4.6. A Brief Workflow of This Study

**Figure 12:**
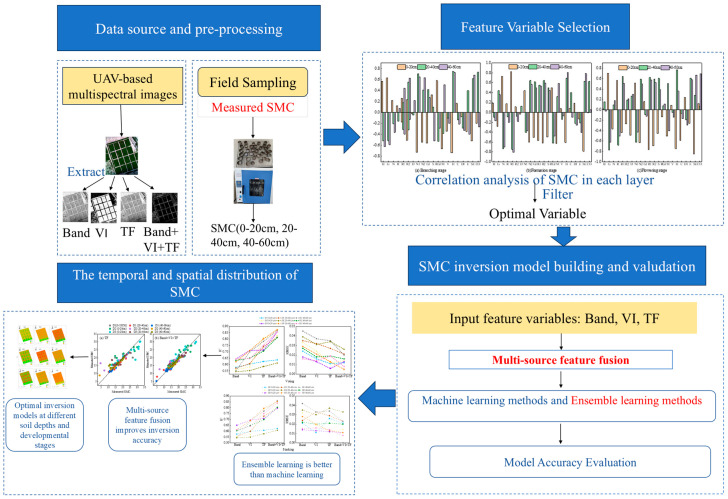
A brief flowchart of the study results and analysis.

The workflow of this study is shown in Figure 12 and includes three parts. (1) Data collection and processing: Soil samples at different growth stages and depths were collected from May to September 2025. After stitching, correcting, and cropping UAV multispectral images, we extracted band reflectance and texture features from the multispectral images, and calculated spectral indices. Next, we performed feature fusion: we combined spectral features (including band reflectance and spectral indices) with texture features into a comprehensive feature vector, usually achieved by direct concatenation or similar methods, to fully leverage the complementarity of multi-source information. During feature fusion, normalization procedures such as min–max normalization were applied to eliminate differences in units among different features, ensuring that all feature values are on the same scale and improving the stability of model training. Finally, the normalized fused features were used as input variables for the model, completing the feature variable fusion process. (2) Variable selection was performed on band reflectance, spectral indices, and texture features using Pearson correlation analysis, and the optimal variables were input into the model for inversion. (3) Result analysis and conclusions: The final results confirmed that the inversion accuracy of the Stacking and Voting ensemble learning models was higher than that of the KNN, RFR, and XG-Boost machine learning models.

## 5. Conclusions

(1) The integration of multi-source features significantly enhances the predictive performance of the model. When using a single-feature variable, the prediction accuracy of soil moisture is relatively low, whereas the performance of three machine learning models and two ensemble learning models improves significantly when we adopt multi-source feature integration.
(2) There are significant differences in the optimal choice of soil moisture inversion depth at different growth stages. During the branching stage and the budding stage, the optimal inversion depth is 40–60 cm, with *R*^2^ values of 0.813 and 0.790 for the validation set, respectively; while at the early flowering stage, the optimal inversion depth is 20–40 cm, with an *R*^2^ of 0.790 for the validation set.
(3) Ensemble learning models showed superior and stable performance in our study, especially the Voting and Stacking models, with maximum *R*^2^ values of 0.874 and 0.853, respectively. Compared to individual machine learning models, ensemble learning improves overall prediction accuracy.

The approach combining multi-source feature fusion and ensemble learning models provides some theoretical support for the development of precision agriculture. However, future research needs to further explore the adaptability of the model under different soil types and climatic conditions and consider a broader range of datasets and prediction scenarios to achieve feasibility and effectiveness for large-scale applications.

## Figures and Tables

**Table 1:** Characteristics of soil moisture changes in alfalfa at different growth stages.

Dataset	Depth (cm)	Observations	Min %	Max %	Mean	SD	CV
branching stage	0–20	48	8.3	26.8	16.1	3.2	19.9
	20–40	48	12.8	29.9	21.4	5.2	24.7
	40–60	48	12.1	31.5	21.9	5.5	25.2
budding stage	0–20	48	5.1	23.7	15.1	4.0	26.7
	20–40	48	4.6	24.5	17.3	4.6	26.4
	40–60	48	12.4	25.13	18.9	6.0	31.7
early flowering stage	0–20	48	4.7	15.7	12.3	2.5	20.1
	20–40	48	3.3	20.1	14.6	4.3	29.3
	40–60	48	10.5	21.3	16.7	3.7	22.2

**Table 2:** The central wavelength and reflectivity of the diffuse reflector.

Spectral Band	Center Wavelength/nm	Bandwidth/nm	Reflectance of Diffuse Reflector/%
Blue	450	35	60
Green	555	25	60
Red	660	20	60
Rededge 1	720	10	60
Rededge 2	750	15	60
NIR	840	35	60

**Table 3:** Spectral index.

Spectral Indices	Acronyms	Formulation	Reference
Normalized difference vegetation index 1	NDVI1	NDVI1 = (G − B)/(G + B)	[48]
Normalized difference vegetation index 2	NDVI2	(NIR − R)/(NIR + R)	[49]
Ratio vegetation index 1	RVI1	RVI1 = NIR/G	[50]
Ratio vegetation index 2	RVI2	NIR/R	[51]
Difference vegetation index	DVI	NIR − R	[52]
Green index	GI	G/R	[53]
Simple ratio pigment index	SRPI	B/R	[54]
Red-to-green ratio index	RGRI	R/G	[55]

## Data Availability

All data are incorporated into the article.

## References

[1] Tian M., Ge X., Ding J., Wang J., Zhang Z. (2020). Soil moisture estimation by coupling machine learning and airborne hyperspectral data. Prog. Laser Optoelectron..

[2] Cheng M., Jiao X., Guo W., Wang S., Pan Y., Zhang H., Sang H. (2020). Temporal and spatial distribution characteristics of irrigation water requirement for main crops in the plain area of Hebei Province. Irrig. Drain..

[3] Yang X., Yu Y. (2017). Remote sensing retrieval of soil moisture based on experimental reflectance spectra. Trans. Chin. Soc. Agric. Eng..

[4] Wang S., Jiao W., Zhao W., Sun D., Lang S., Jia Y., Zhang H., Xu C. (2026). Detecting multiple types of marine floating debris via satellite remote sensing informed by hyperspectral features. Ecol. Indic..

[5] Gallego P.M., Fernandez I.D., Marston C. (2026). The application of satellite remote sensing to coastal dune environments: A systematic review. Prog. Phys. Geogr. Earth Environ..

[6] Chen J., Cui Z., Tang Z. (2026). Edge effects on forest dynamics in China from 2000 to 2020: Evidence from satellite remote sensing. Remote Sens. Environ..

[7] Chen T., Zhuo G., La B. (2017). MODIS-based remote sensing retrieval of soil moisture in eastern Nagqu. Soil Bull..

[8] Liu J., Zhao C., Yang G., Yu H., Zhao X., Xu B., Niu Q. (2016). Advances in UAV remote sensing for extracting field crop phenotypic information. Trans. Chin. Soc. Agric. Eng..

[9] Alahmad T., Nemenyi M., Szeles A., Ali N., Hijazi O., Nyeki A. (2025). Spatiotemporal prediction of soil moisture content at various depths in three soil types using machine learning algorithms. Front. Soil Sci..

[10] Wang Y., Ma Y., Fan X., Chen H., Hu X. (2025). Estimation model of a comprehensive water index for summer maize based on UAV multispectral data. Trans. Chin. Soc. Agric. Mach..

[11] Jin Y., Wu X., Zhen W., Cui X., Chen L., Qie Z. (2024). Soil moisture retrieval from UAV multispectral imagery based on optimization of sampling-point spectral information window scale. Trans. Chin. Soc. Agric. Mach..

[12] Fei S., Hassan M.A., He Z., Chen Z., Shu M., Wang J., Li C., Xiao Y. (2021). Assessment of ensemble learning to predict wheat grain yield based on UAV-multispectral reflectance. Remote Sens..

[13] Peppes N., Daskalakis E., Alexakis T., Adamopoulou E., Demestichas K. (2021). Performance of machine learning-based multi-model voting ensemble methods for network threat detection in Agriculture 4.0. Sensors.

[14] Das B., Rathore P., Roy D., Chakraborty D., Jatav R.S., Sethi D., Kumar P. (2022). Comparison of bagging, boosting and stacking algorithms for surface soil moisture mapping using optical-thermal-microwave remote sensing synergies. CATENA.

[15] Shen Z., He Q., Yang C., Cheng Z. (2026). Soil moisture retrieval under different land-cover conditions based on Sentinel-1 SAR. Remote Sens. Environ..

[16] Teal R.K., Tubana B., Girma K., Freeman K.W., Arnall D.B., Walsh O., Raun W.R. (2006). In-season prediction of corn grain yield potential using normalized difference vegetation index. Agron. J..

[17] Wang S., Li R., Wu Y., Zhao S., Wang X. (2022). Multi-model integrated inversion of surface soil moisture based on model averaging. Trans. Chin. Soc. Agric. Eng..

[18] Luo Y., Xu Q., Guo Y., Sun Z., Jin Y., Chen J., Yu W. (2023). Study on the Effect of Scale Effect on Soil Moisture Monitoring by Multispectral Remote Sensing. Water-Sav. Irrig..

[19] Bian M., Ma Y., Fan Y., Chen Z., Yang G., Feng H. (2023). Estimation of potato chlorophyll content by integrating UAV multi-source sensors. Trans. Chin. Soc. Agric. Mach..

[20] Greene S.L., Kesoju S.R., Martin R.C., Kramer M. (2015). Occurrence of transgenic feral alfalfa (*Medicago sativa* subsp. *sativa* L.) in alfalfa seed production areas in the United States. PLoS ONE.

[21] Zhang A.W., Yin S.N., Wang J., He N., Chai S., Pang H. (2023). Grassland chlorophyll content estimation from drone hyperspectral images combined with fractional-order derivative. Remote Sens..

[22] Bagavathiannan M.V., Gulden R.H., Begg G.S., Acker R.C.V. (2010). The demography of feral alfalfa (*Medicago sativa* L.) populations occurring in roadside habitats in Southern Manitoba, Canada: Implications for novel trait confinement. Environ. Sci. Pollut. Res..

[23] Xu L., Xu D., Pang H., Xin X., Jin D., Tang X., Guo M. (2017). Suitability zoning of Medicago species in China. Grassl. Sci..

[24] Wang C., Tian W., Yang Y., Lian H., Wang Z. (2004). Domestic and international introduction trials of 10 alfalfa (*Medicago sativa*) cultivars. J. Northwest AF Univ. (Nat. Sci.).

[25] Lee J., Ryu Y., Kim J., Dechant B., Lee S., Lim J. (2026). Development of an automated hyperspectral system to monitor hyperspectral reflectance and sun-induced chlorophyll fluorescence with directional and hemispheric view geometries simultaneously. Remote Sens. Environ..

[26] Zhang N., Hong Y., Qin Q., Liu L. (2013). VSDI: A visible and shortwave infrared drought index for monitoring soil and vegetation moisture based on optical remote sensing. Int. J. Remote Sens..

[27] Tsuchiya Y., Hirono Y., Seki H., Sonobe R. (2025). Chlorophyll and carotenoid content estimation using hyperspectral reflectance data from a compact spectrometer and fractal-order derivative analysis. Int. J. Remote Sens..

[28] Bao Y.S., Yan J., Min J.Z., Wang D.M., Li Z.T., Li X.C. (2014). Agricultural drought monitoring in the northern Huaibei region of Jiangsu Province based on the temperature–vegetation drought index. Trans. Chin. Soc. Agric. Eng..

[29] Wang J.X., Pan Y.Z., Zhu X.F., Sun Z.L. (2019). A Review of Researches on Inversion of Eigenvariance of Soil Water. Acta Pedologica Sinica.

[30] Lu B., Dao P.D., Liu J., He Y., Shang J. (2020). Recent advances of hyperspectral imaging technology and applications in agriculture. Remote Sens..

[31] Rischbeck P., Elsayed S., Mistele B., Barmeier G., Heil K., Schmidhalter U. (2016). Data fusion of spectral, thermal and canopy height parameters for improved yield prediction of drought-stressed spring barley. Eur. J. Agron..

[32] Zhou Y.Q., Wang D.L., Chen C., Li R., Li D.S., Liu T., Sun C.M., Zhong X.C., Liu S.P., Ding D.W. (2021). Wheat yield prediction based on color and texture feature indices of UAV RGB images. J. Yangzhou Univ. (Agric. Life Sci.).

[33] Ma Y., Ma L., Zhang Q., Huang C.P., Yi X., Chen X.Y., Hou T.Y., Lv X., Zhang Z. (2022). Cotton yield estimation based on vegetation indices and texture features derived from RGB image. Front. Plant Sci..

[34] Sun J., Jin X.M., Mao H.P., Wu X.H., Zhu W.J., Zhang X.D., Gao H.Y. (2014). Detection of lettuce nitrogen content based on spectral and texture information of hyperspectral images. Trans. Chin. Soc. Agric. Eng..

[35] Lundberg S.M., Erion G., Chen H., Degrave A., Prutkin J.M., Nair B., Katz R., Himmelfarb J., Bansal N., Lee S.I. (2020). From local explanations to global understanding with explainable AI for trees. Nat. Mach. Intell..

[36] Lundberg S.M., Lee S.I. A unified approach to interpreting model predictions. Proceedings of the 31st Annual Conference on Neural Information Processing Systems (NeurIPS).

[37] Maimaitijiang M., Sagan V., Sidike P., Hartling S., Esposito F., Fritschi F.B. (2020). Soybean yield prediction from UAV using multimodal data fusion and deep learning. Remote Sens. Environ..

[38] Li K.X., Zhou S.L., Yin C.Q., Ye Y.B., Han X.Y., Sun S.M. (2025). Estimation of Chlorophyll Content in Cotton Leaves by Fusing UAV Spectral Information and Texture Features. J. Water-Sav. Irrig..

[39] Li H., Huang C., Zhang Y., Li S., Liu Y., Yang K., Lu J. (2025). Estimating maize leaf area index using multi-source features derived from UAV multispectral imagery and machine learning models. Plants.

[40] Sun Z., Yan A., Zhang Z.F., Hou Z.Q., Yuan C., Qu Z.H., Liu P. (2025). Estimation model of maize leaf area index by fusing UAV multispectral and texture features. Jiangsu Agric. Sci..

[41] Wu W. (2023). Study on Water and Fertilizer Effects and UAV Spectral Monitoring Model for Growth Parameters of Sugarcane. Master’s Thesis.

[42] Nopour R. (2025). Development of a prediction model for peptic ulcer disease using the fusion of a feature selection strategy and ensemble algorithms. BMC Gastroenterol..

[43] Yang C.H. (2023). Application of Machine Learning Methods in Estimating the Biomass of Main Economic Crab Species in Zhoushan Fishery. Master’s Thesis.

[44] Singh A., Nawayseh N., Doyon-Poulin P., Milosavljevic S., Rakheja S., Kumar Y., Dewangan K.N., Trask C., Samuel S. (2026). Multi-model machine learning for predicting tractor operator discomfort caused by whole-body vibration. Comput. Electron. Agric..

[45] Feng L., Zhang Z., Ma Y., Du Q., Luck B. (2020). Alfalfa yield prediction using UAV-based hyperspectral imagery and ensemble learning. Remote Sens..

[46] Ge H., Ma F., Li Z., Tan Z., Du C. (2021). Improved accuracy of phenological detection in rice breeding by using ensemble models of machine learning based on UAV-RGB imagery. Remote Sens..

[47] Zhang H.M., Chen L.J., Liu W., Han W.T., Zhang Z.Y., Zhang F. (2021). Estimation of Summer Corn Fractional Vegetation Coverage Based on Stacking Ensemble Learning. Trans. Chin. Soc. Agric. Mach..

[48] Verrelst J., Schaepman M.E., Koetz B., Kneubühler M. (2008). Angular sensitivity analysis of vegetation indices derived from CHRIS/PROBA data. Remote Sens. Environ..

[49] Rouse J.W., Haas R.W., Schell J.A., Deering D.W. (1974). Monitoring the Vernal Advancement and Retrogradation (Greenwave Effect) of Natural Vegetation.

[50] Xue L.H., Cao W.X., Luo W.H., Dai T.B., Zhu Y. (2004). Monitoring leaf nitrogen status in rice with canopy spectral reflectance. Agron. J..

[51] Mishra S., Mishra D.R. (2011). Normalized difference chlorophyll index: A novel model for remote estimation of chlorophyll-a concentration in turbid productive waters. Remote Sens. Environ..

[52] Roujean J.L., Breon F.M. (1995). Estimating PAR absorbed by vegetation from bidirectional reffectance measurements. Remote Sens. Environ..

[53] Zarco-Tejada P.J., Berjon A., Lopez-Lozano R., Miller J.R., Martin P., Cachorro V., Gonzalez M.R., de Frutos A. (2005). Assessing vineyard condition with hyperspectral indices: Leaf and canopy reflectance simulation in a row-structured discontinuous canopy. Remote Sens. Environ..

[54] Zhang Z.T., Tan C.X., Xu C.H., Chen S.B., Han W.T., Li Y. (2019). Study of maize root-zone soil moisture using UAV multispectral remote sensing. Trans. Chin. Soc. Agric. Mach..

[55] Hao J. (2023). Research on Soil Moisture Detection Based on UAV Multispectral imagery. Master’s Thesis.

[56] Dai T., Chen J., Liu H., Liu L., Fan S., Bai X., Qian L., Liu H., Ba Y., Da Q. (2020). Research on the potential of reconstructing spectral indices and dividing crop growth stages based on satellite remote sensing for monitoring soil moisture in farmland. Comput. Electron. Agric..

[57] Kasim N., Sawut R., Shi Q., Maihemuti B. (2018). Estimation of soil organic matter content based on optimized spectral index. Trans. Chin. Soc. Agric. Mach..

[58] Jin Y.H., Wu X.M., Zhen W.C., Cui X.T., Chen L., Qie Z.H. (2024). Unmanned aerial vehicle multispectral remote sensing inversion of soil water content based on the optimisation of spectral information window scale at sampling points. J. Agric. Mach..

